# Non-Hodgkin Lymphoma and Tuberculosis Coexisting in the Same Cervical Lymph Node: A Case Report

**DOI:** 10.7759/cureus.98957

**Published:** 2025-12-11

**Authors:** Abdallah Taha, Sara Alzhrani, Nawaf Aljafari, Bashar A Aljadani, Abdullah Al-Rashdi, Abdulrahman A Almuntashiri, Khalid M Alkhalifah, Wafa Bahshwan

**Affiliations:** 1 General Surgery Department, Faculty of Medicine, South Valley University, Qena, EGY; 2 Medicine, Umm Al-Qura University, Al Qunfudah, SAU; 3 General Surgery Department, Al Qunfudah General Hospital, Al Qunfudah, SAU; 4 Medicine and Surgery, College of Medicine, Umm Al-Qura University, Jeddah, SAU; 5 Otorhinolaryngology - Head and Neck Surgery, Security Forces Hospital, Riyadh, SAU; 6 Makkah Health Cluster, Ministry of Health, Al Qunfudah, SAU

**Keywords:** cervical lymph node, lymphoma, mycobacterium tuberculosis, non-hodgkin, tuberculosis

## Abstract

We report the case of a 60-year-old female from Saudi Arabia who presented with a six-month history of a neck mass. Following an excisional biopsy under local anesthetic, laboratory analyses, including polymerase chain reaction (PCR), tuberculosis culture, and microscopic tissue examination, revealed the presence of diffuse large B-cell lymphoma (DLBCL) alongside tuberculous lymphadenitis in the cervical region. Immunohistochemistry confirmed DLBCL with CD20+, BCL6+, MUM1+, and Ki-67 80%. The patient was managed sequentially with anti-tuberculous therapy (ATT) followed by R-CHOP (rituximab, cyclophosphamide, doxorubicin, vincristine, prednisone) regimen, achieving complete remission at six months with 12-month disease-free follow-up. This report highlights that lymphoma and tuberculous lymphadenitis can coexist. In patients undergoing lymph node biopsy for suspected tuberculosis, it is crucial to thoroughly assess for an underlying lymphoma. Detecting a malignancy in a cervical tuberculous lymph node significantly alters the therapeutic approach and requires coordinated management with medical oncology specialists.

## Introduction

Tuberculosis (TB) is a chronic infectious disease caused by Mycobacterium tuberculosis, with its development and recurrence often promoted by impaired cell-mediated immunity [1]. The most common clinical manifestations of tuberculous lymphadenitis include enlarged cervical lymph nodes, often accompanied by systemic symptoms such as fever, weight loss, and night sweats [2]. In contrast, non-Hodgkin lymphoma (NHL) may arise following chronic inflammatory conditions and is also associated with immune system impairment [3]. The earliest reported association between TB and carcinoma dates back nearly 200 years, when Bayle described cancerous cavitation as a form of TB [4]. 

The complex relationship between TB and cancer has been increasingly recognized, particularly in countries where TB remains highly prevalent [5]. However, the exact nature of this interaction is not yet fully understood. Their co-occurrence may be coincidental. Some researchers have suggested that cachexia associated with cancer could provide a favorable environment for latent mycobacteria. Conversely, cancer may develop in patients with long-standing, progressive TB [6]. Prolonged immune suppression may further create conditions conducive to the concurrent or sequential development of both diseases in the same individual [7]. 

While TB is primarily controlled by T-cell-mediated immunity, diffuse large B-cell NHL arises from malignant B-cells. Some reports have described cases in which malignant lymphoma and TB occur simultaneously in the same patient [8,9]. Nevertheless, only a few cases have specifically described the coexistence of these conditions within the cervical lymph nodes [10]. In 1855, Rokitansky proposed that TB and malignancy could not coexist within the same organ, suggesting an antagonistic relationship between the two [6]. However, in 1899, Whartin documented the first case of TB and cancer occurring together in the same enlarged lymph node. This phenomenon remains rare, with only a limited number of cases reported in the literature, particularly in regions with a high TB burden [6,11,12]. 

In Saudi Arabia, TB continues to pose a considerable public health issue, with an estimated incidence of roughly 10-12 cases per 100,000 individuals. At the same time, lymphoma is among the most frequently occurring hematologic cancers, with NHL representing approximately 4-5% of all new cancer diagnoses [13,14]. This report presents a case of NHL and TB coexisting within the same cervical lymph node. It underscores the potential diagnostic challenges posed by the simultaneous presence of NHL and TB, which may delay appropriate diagnosis and treatment of either condition.

## Case presentation

A 60-year-old Saudi female sought treatment at Alqunfudah General Hospital for a progressively painful and growing mass on the left side of her neck. The mass was associated with upper abdominal pain and intermittent fever for six months. Initially, it had been small and painless, but it had gradually increased in size, become tender to palpation, and caused dysphagia, prompting her to seek medical care. Her past medical history was notable for chronic kidney disease and hypertension. She had no history of tuberculosis or malignancy, and her family history was unremarkable.

On examination, the patient was conscious and oriented but appeared mildly distressed due to pain. Vital signs were within normal limits, except for a low-grade fever. Local examination revealed a firm, irregular, slightly tender mass with limited mobility, measuring approximately 5 × 5 cm, on the left side of the neck. The overlying skin was erythematous, but no ulceration was present. No palpable lymphadenopathy was detected in the axillary, supraclavicular, inguinal, or popliteal regions. The remainder of the systemic examination was unremarkable.

Laboratory tests, including a complete blood count (CBC), showed an elevated white blood cell count (13,900/µL; normal: 4,000-11,000/µL) with a predominance of neutrophils (normal: 40-60%). Markers of inflammation, specifically the erythrocyte sedimentation rate (ESR) and C-reactive protein (CRP), were also high (ESR: 58 mm/hr; normal: ≤15 mm/hr for men, ≤20 mm/hr for women) and (CRP: 42 mg/L; normal: <10 mg/L). Lymphoma-related labs included elevated LDH (635 U/L; normal: 140-280 U/L), normal uric acid (4.1 mg/dL; normal: 2.4-6.0 mg/dL), and elevated β2-microglobulin (3.5 mg/L; normal: 0.7-1.8 mg/L). Markers for cell turnover, such as LDH and β2-microglobulin, were assessed as part of the lymphoma workup, indicating high tumor burden; no evidence of tumor lysis syndrome was observed. 

**Figure 1:**
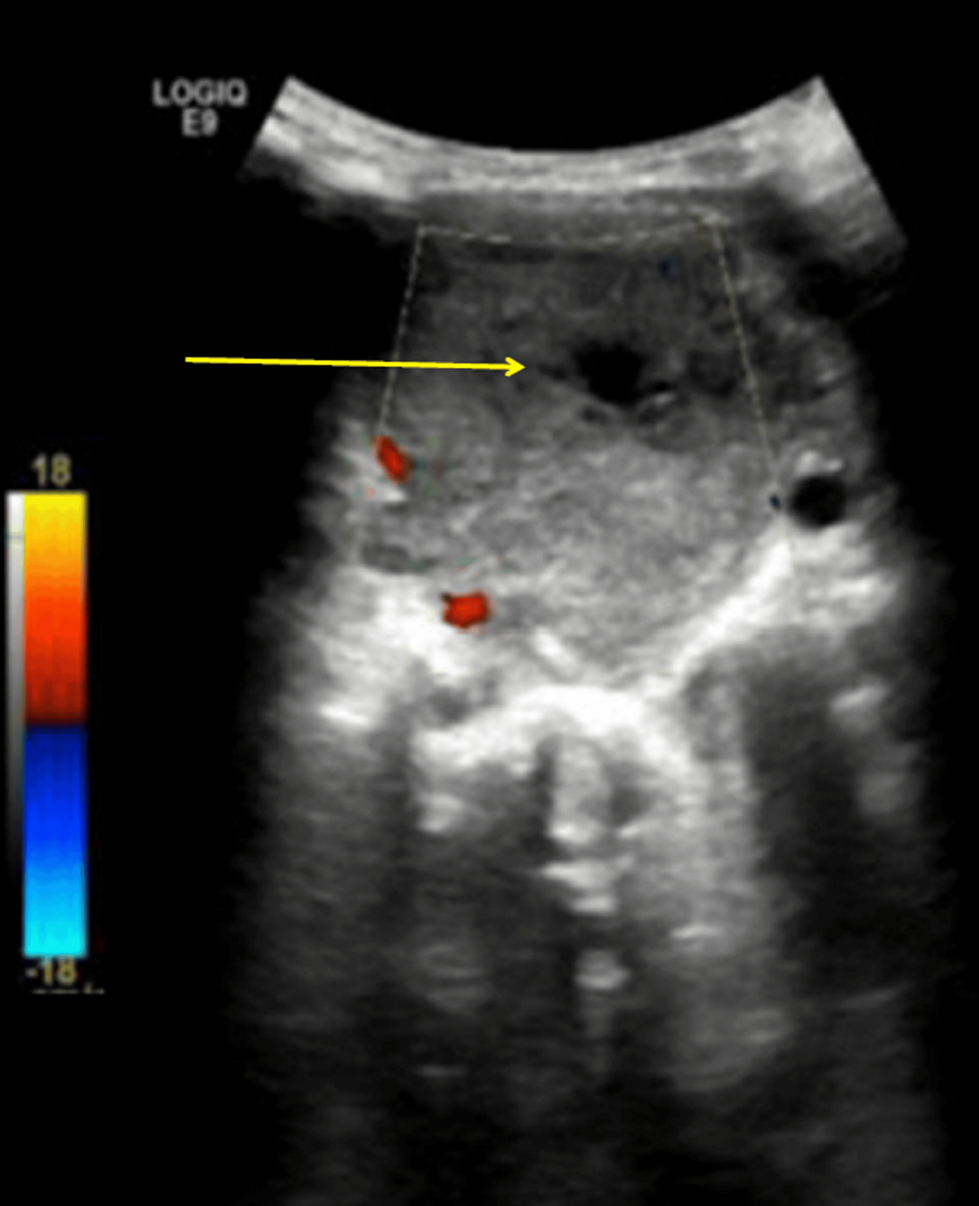
Ultrasound of the neck showing a heterogeneous solid mass with a necrotic center (yellow arrow)

Ultrasound examination of the neck revealed a heterogeneous solid mass with central necrosis, measuring approximately 5.4 × 4.9 cm, located on the left side, with significant internal vascularity (Figure 1).

**Figure 2:**
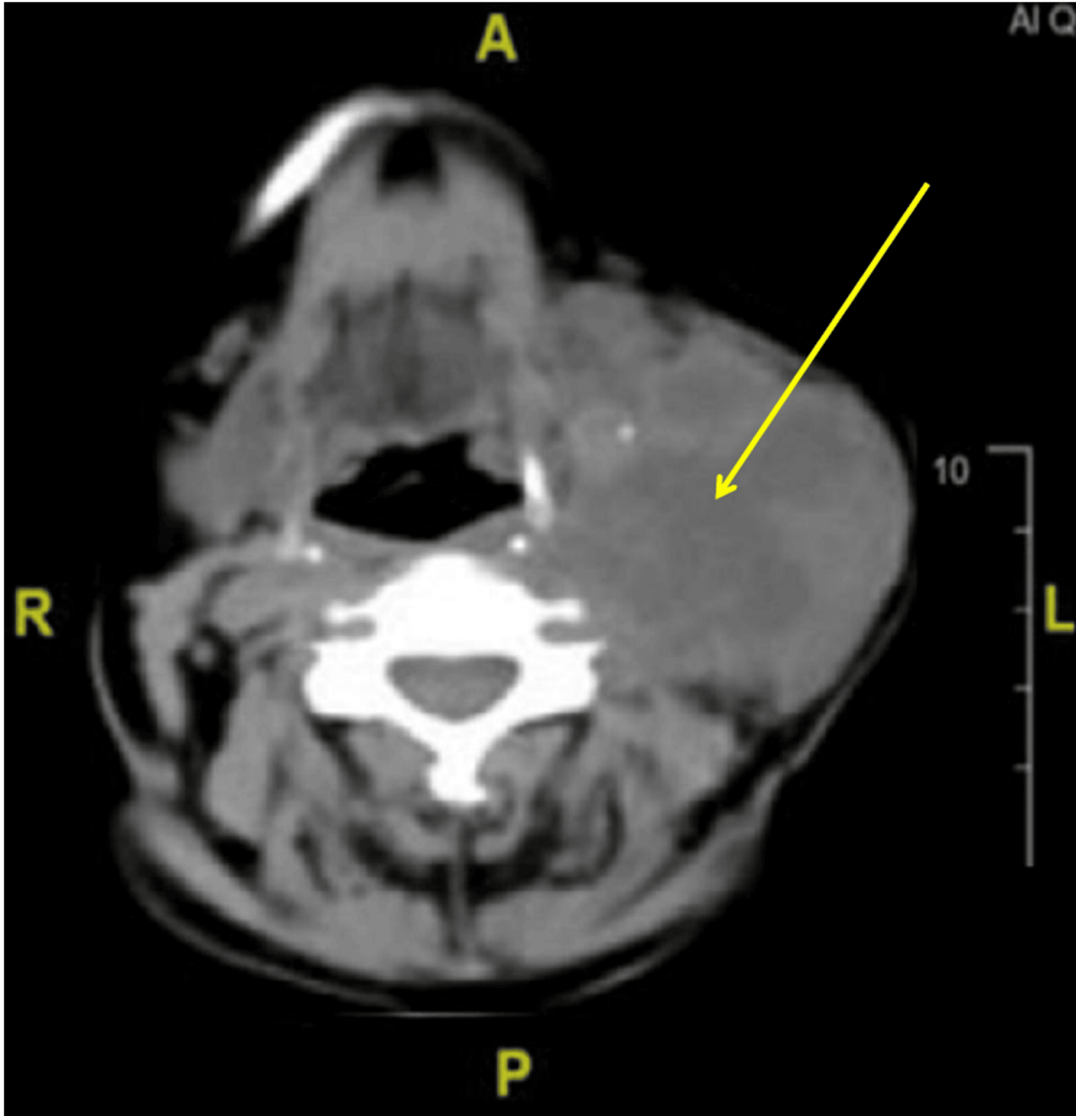
Contrast-enhanced CT of the neck showing a heterogeneous solid mass in the left lateral neck (yellow arrow) CT: computed tomography

A contrast-enhanced CT scan of the neck demonstrated a heterogeneous solid mass measuring 5.6 × 5.7 × 5.1 cm (longitudinal superior × anteroposterior × transverse sagittal) in the left lateral neck, deep to the sternocleidomastoid muscle. The mass extended from the C3 to C6 vertebral levels. Adjacent tissue planes were poorly defined, with lateral displacement of the sternocleidomastoid muscle and medial displacement of the left common carotid artery and internal jugular vein (Figure 2).

An incisional biopsy was performed under local anesthesia with mild sedation. A transverse cervical incision was made over the lateral neck directly above the mass. Multiple small tissue fragments were obtained from the firm, matted lesion. Samples were submitted for different analyses: one in saline for TB polymerase chain reaction (PCR) and culture, another in 10% formalin for histopathology, and a third for bacterial culture and sensitivity.

PCR testing for Mycobacterium TB was positive. Histopathological examination revealed features of tuberculous lymphadenitis along with evidence of high-grade NHL, specifically diffuse large B-cell lymphoma (DLBCL) subtype. Immunohistochemistry (IHC) markers were done to confirm the diagnosis, revealing malignant lymphoid cells positive for CD20 (diffuse membranous staining), BCL6 (nuclear positivity in >30% of cells), and MUM1 (post-germinal center phenotype), with a high Ki-67 proliferation index of 80%, consistent with activated B-cell subtype DLBCL. CD3 and CD5 were negative, ruling out T-cell lymphoma, and CD10 was weakly positive in a subset of cells. Based on these findings, the patient was referred to the infectious disease and medical oncology departments for integrated management of both conditions. 

The patient was managed with a sequential approach, prioritizing TB control. ATT was initiated first: a two-month intensive phase with isoniazid (5 mg/kg), rifampin (10 mg/kg), pyrazinamide (25 mg/kg), and ethambutol (15 mg/kg), followed by a four-month continuation phase with isoniazid and rifampin. The patient tolerated ATT well, with resolution of fever and partial reduction in lymph node size within four weeks, though mild gastrointestinal upset was noted and managed symptomatically. After four weeks of ATT, with confirmed negative sputum cultures and clinical improvement, R-CHOP chemotherapy (rituximab 375 mg/m², cyclophosphamide 750 mg/m², doxorubicin 50 mg/m², vincristine 1.4 mg/m², and prednisone 100 mg daily for five days) was initiated for six cycles every 21 days.

The patient tolerated chemotherapy moderately, experiencing grade 2 neutropenia (managed with G-CSF) and mild peripheral neuropathy. She showed a partial response after three cycles, with a 50% reduction in lymph node size on follow-up CT, and achieved complete remission at six months post-diagnosis. Follow-up duration was 12 months, during which the patient remained disease-free but required ongoing monitoring for chronic kidney disease.

## Discussion

We describe an uncommon and diagnostically challenging scenario in which DLBCL was identified concurrently with tuberculous lymphadenitis within the same cervical lymph node. This unusual coexistence raises important clinical questions regarding disease pathogenesis, the risk of diagnostic pitfalls, and optimal treatment strategies. 

Tuberculous lymphadenitis is one of the most common forms of extrapulmonary TB, particularly in regions where the disease is endemic [15]. Clinically, it often presents as a slowly enlarging, painless mass that may later become tender or suppurative, as seen in our patient. The presence of systemic symptoms such as fever, weight loss, and elevated inflammatory markers (ESR, CRP) further supported the suspicion of an infectious cause. A similar case has been reported where tuberculous lymphadenitis was found to coexist with diffuse large B-cell lymphoma [16], highlighting the clinical difficulty in differentiating between the two conditions. However, the simultaneous development of DLBCL complicated the diagnosis, as lymphomas can also present with lymph node enlargement and systemic manifestations [17]. Although TB lymphadenitis is well recognized, its coexistence with lymphoma - particularly DLBCL - is rare but has been reported.

Chronic inflammation due to persistent infections, including TB, has been implicated in lymphomagenesis through mechanisms such as sustained antigenic stimulation and immune dysregulation [18]. Chronic TB infection may be linked to B-cell transformation through several immunologic mechanisms. Persistent Mycobacterium TB antigens (e.g., ESAT-6, CFP-10) are processed by antigen-presenting cells (macrophages) in the lymph node, activating CD4+ T-cells via MHC class II presentation and releasing IFN-γ and TNF-α. Cytokines recruit and activate B-cells, leading to germinal center formation and polyclonal B-cell expansion. Chronic stimulation causes somatic hypermutation and class-switch recombination in B-cells, increasing the risk of oncogenic mutations (e.g., translocations involving BCL6). Impaired regulatory T-cells (due to TB-induced exhaustion) fail to suppress this, allowing clonal selection. Transformed B-cells evade apoptosis through upregulated anti-apoptotic proteins (e.g., BCL2), culminating in DLBCL. In immunocompromised states (e.g., due to chronic kidney disease), this amplifies the risk.

**Figure 3:**
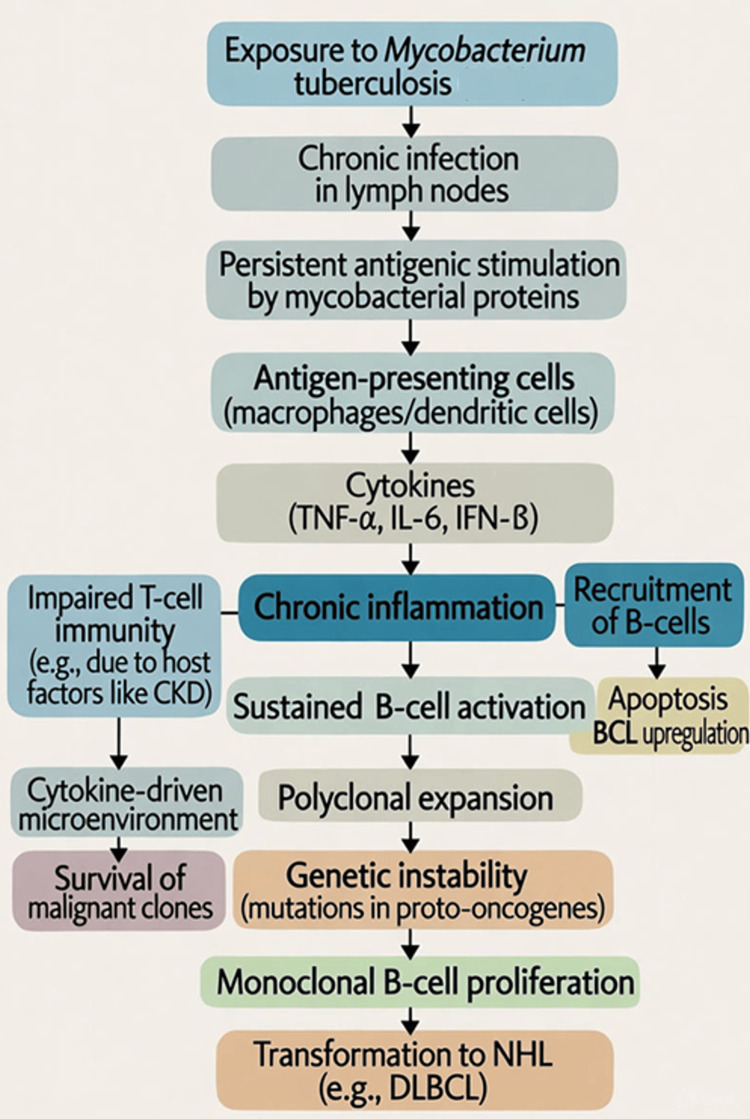
Hypothetical immunopathogenic sequence from chronic TB to DLBCL development Exposure to Mycobacterium tuberculosis leads to chronic lymph node infection and persistent antigenic stimulation, triggering cytokine release (TNF-α, IL-6, IFN-γ), B-cell recruitment, and polyclonal expansion. Impaired T-cell immunity (e.g., due to CKD) and genetic instability culminate in monoclonal B-cell transformation to DLBCL, enhanced by BCL2-mediated apoptosis inhibition in the cytokine-driven microenvironment TB: tuberculosis; DLBCL: diffuse large B-cell lymphoma; TNF-α: tumor necrosis factor-alpha; IL-6: interleukin 6; IFN-γ: interferon-gamma; CKD: chronic kidney disease

The treatment and follow-up in this case align with the above-mentioned mechanisms, as initiating ATT first addressed chronic antigenic stimulation, reducing the inflammatory cytokine milieu that could perpetuate B-cell transformation. The flowchart in Figure 3 summarizes a hypothetical Immunopathogenic sequence from chronic TB to DLBCL development. Sequential R-CHOP then targeted the malignant B-cells, preventing further lymphoproliferation. The 12-month disease-free follow-up supports early intervention in disrupting this sequence. 

This case demonstrates both similarities and differences when compared with previously reported cases [19]. Our patient presented with progressive cervical lymphadenopathy and systemic features, resembling other documented reports. However, her comorbid chronic kidney disease may have contributed to the simultaneous occurrence of both conditions, highlighting the influence of host factors on disease presentation. In contrast, Karleen and Saniasiaya’s report of a 26-year-old male showed more acute inflammatory signs, including erythema and fluctuance [20], while Lemus and Revelo described a 46-year-old female with a more indolent course typical of TB lymphadenitis [21]. Such variations underscore the diagnostic challenge of differentiating between infectious, inflammatory, and malignant causes of cervical lymphadenopathy.

Diagnostic strategies in these cases also differ. In our patient, the diagnosis was achieved at the initial assessment through PCR testing and histopathology. In contrast, Lemus and Revelo’s case [21] required a repeat biopsy after inconclusive initial results. This emphasizes the importance of persistent investigation in unexplained lymphadenopathy. Karleen and Saniasiaya’s report [20], which lacked definitive diagnostic detail, illustrates how easily lymphoma or granulomatous infections may be misdiagnosed as benign inflammatory processes. Histopathology in our case demonstrated both granulomatous inflammation and malignant lymphoid proliferation, strongly supporting the hypothesis of a possible link between chronic TB infection and lymphoma development, consistent with prior literature [18,21]. 

Managing these complex cases requires careful consideration of how the diseases interact and the priority of treatments. The patient’s management adhered well to guidelines from the WHO for TB and the National Comprehensive Cancer Network (NCCN) for DLBCL. WHO recommends prioritizing ATT in co-infected cases to prevent dissemination, which was followed here with a four-week lead time before R-CHOP, mirroring NCCN's emphasis on infection control before immunosuppression. The regimen choices (standard ATT and R-CHOP) and monitoring for interactions (e.g., rifampin effects on rituximab) were appropriate, though closer immune reconstitution inflammatory syndrome (IRIS) surveillance could have been emphasized. Overall, the multidisciplinary approach and complete remission outcome indicate effective guideline compliance, superior to cases with diagnostic delays leading to poorer prognosis.

Unlike the case reported by Lemus and Revelo [21], which was successfully treated with only ATT medication, our patient’s dual diagnosis necessitated a coordinated treatment plan involving specialists in infectious diseases and cancer to manage both conditions effectively. This approach aligns with the current understanding that untreated TB needs to be addressed before starting chemotherapy that suppresses the immune system, to prevent the spread of TB or the development of IRIS. The difference between these cases illustrates how treatment strategies must be adapted to the specific pathological findings and individual patient risk factors. 

These comparative observations lead to several important clinical conclusions. Firstly, they emphasize the need to obtain sufficient tissue samples for both microscopic examination and microbiological analysis in cases of persistent lymph node enlargement. Secondly, they highlight how patient characteristics such as age and immune status can significantly impact how a disease manifests and progresses. Lastly, they demonstrate the crucial role of collaboration among different medical specialties in managing complex cases where multiple disease processes occur together. The insights gained from this comparative analysis contribute to a better understanding of the diagnostic and therapeutic challenges presented by the simultaneous occurrence of infectious and cancerous lymph node pathology. 

The definitive diagnosis in this case was established through microscopic examination of the tissue and PCR testing. Typically, TB lymphadenitis is characterized by granulomas with central necrosis, while DLBCL shows large, abnormal B-cells with a high rate of cell division [22]. The simultaneous presence of both these features underscores the significance of obtaining adequate tissue samples-sending specimens for TB PCR, culture, and microscopic analysis was vital in preventing a missed diagnosis [16]. 

Treating TB and DLBCL at the same time requires a multidisciplinary approach. The TB infection must be addressed first to prevent it from spreading, particularly before starting chemotherapy that weakens the immune system [23]. However, delaying treatment for lymphoma can allow cancer to progress. Current guidelines suggest initiating (ATT) followed by R-CHOP once the infection is under control [24]. Close monitoring for interactions between medications (e.g., rifampin potentially reducing the effectiveness of rituximab) and for IRIS is crucial [25]. 

The long-term outlook for DLBCL that develops alongside TB lymphadenitis remains uncertain due to the rarity of this occurrence. However, research suggests that diagnosing both conditions early and accurately improves patient outcomes [17]. Factors such as the patient’s other health conditions (chronic kidney disease, hypertension) and potential side effects from chemotherapy must be carefully considered [24].

## Conclusions

Lymphoma and tuberculous lymphadenitis can occur simultaneously within the same lymph node. It is essential to carefully investigate for the potential presence of lymphoma in lymph node biopsies from individuals with suspected TB. Identifying a malignancy in a cervical tuberculous lymph node biopsy necessitates a substantial modification of the treatment strategy, requiring the involvement of medical oncology. In TB-endemic regions, persistent lymphadenopathy warrants histopathologic and microbiologic evaluation to rule out dual pathology. Early recognition and timely multidisciplinary collaboration are crucial to improving outcomes in such complex cases. Greater awareness among clinicians can help reduce diagnostic delays and optimize patient care. Future reports and case accumulations may provide further insights into the best diagnostic and therapeutic approaches for patients presenting with dual pathologies.
